# Emergence of Neural Face Selectivity in Infants Younger Than 4 Months Old

**DOI:** 10.1111/infa.70076

**Published:** 2026-02-21

**Authors:** Diane Rekow, Tanisha Arya, Duygu H. Bayir, Brigitte Röder

**Affiliations:** ^1^ Biological Psychology and Neuropsychology University of Hamburg Hamburg Germany; ^2^ Crossmodal Perception and Plasticity Laboratory Institute of Research in Psychology (IPSY) and Institute of Neuroscience (IoNS) Universite Catholique de Louvain Louvain‐la‐Neuve Belgium; ^3^ LV Prasad Eye Institute Hyderabad India

**Keywords:** face categorization, face perception, frequency‐tagging EEG, low‐acuity vision, rapid visual stimulation

## Abstract

Research on face‐selectivity in infants under 4 months has shown mixed results, especially with rapid stimulus presentations. Here we tested whether increasing face saliency would promote face‐selective responses even with brief presentation times in infants aged 4‐to‐6 months and younger. Using frequency‐tagging EEG, we presented face and nonface stimuli at a rapid 6‐Hz rate (i.e., 167 ms/stimulus), with faces appearing once per second as every 6th stimulus, therefore isolating face‐selectivity at 1 Hz in the EEG spectrum. Two sets of images were presented in separate conditions: a “classic” set from previous studies and a “new” more salient set with increased luminance and size of the depicted items as well as a smoother background intending to facilitate figure‐ground segregation. In Experiment 1, we validated the use of the new set to elicit high‐level face‐selectivity in adults (*N* = 19). Crucially, Experiment 2 demonstrated benefits from the new set for 2‐to‐6‐month old infants (*N* = 46), with the youngest ones (2‐to‐4‐month‐olds) featuring face‐selective responses only to this new set. Thus, adapting stimuli to the visual capabilities of infants uncovered earlier developmental emergence of face‐selectivity to rapid visual stimulation than previously thought.

## 1 Introduction

Infant high‐level visual perception has been the target of developmental studies for decades, yet, the emergence of category‐selective activity in the young human brain remains undetermined. Face perception follows a protracted developmental trajectory (Pascalis et al. 2011), and neural face‐selectivity—reflecting a preferential (i.e., stronger) neural response to faces compared to other visual categories—was long considered to emerge, at best, late in the first year of life (Ayzenberg and Behrmann 2024). Recent functional magnetic resonance imaging (fMRI) studies in young infants (Deen et al. 2017; Kosakowski et al. 2024) pointed toward an earlier existence of face‐selective activity, in typical sites of the mature face network, for example the fusiform face area in the fusiform gyrus (FFA (Kanwisher et al. 1997) or the superior temporal sulcus (STS (Allison et al. 2000)), notably involved in the perception of moving faces. These findings gave insight into the early scaffolding of visual cognition through category‐selectivity from 2 months old. However, although these studies used video clips which presented real faces from different viewpoints and expressiveness levels, they employed the same limited series of stimuli, weakening the generalizability of the target category (e.g., 18 video clips of 8 children faces in total in Kosakowski et al. (2024)), as well as its selectivity through limited contrasts to other categories (Deen et al. 2017). In real life, visual cognition develops while not all visual inputs allow long fixations, and perception largely occurs at a glance, that is, within a single fixation. From already 2 months old, infant's saccades are actually as quick as those of adults (Garbutt et al. 2006), suggesting that their exploration of their visual environment is able to react to quickly sampled visual inputs. Thus, it remains unclear whether the face‐selectivity observed from 2 months of age generalizes across a larger number of variable stimuli (i.e., both within the face category and across multiple nonface categories), and whether face selectivity would unfold under tight temporal constraints.

To mimic the high diversity of faces and the visual constraints we are exposed to in real life, Rossion et al. (2015) developed a fast periodic visual stimulation (FPVS) paradigm using electroencephalography (EEG) to assess the adult's categorization of faces at a glance. These FPVS sequences were of short durations (i.e., 60 s), relied on the very brief (i.e., 167 ms) presentation time of stimuli and had nonface objects interleaved with a periodic occurrence of faces to *tag* a face‐selective neural activity at a predefined frequency (i.e., frequency‐tagging EEG or FT‐EEG). Importantly in this case, FT‐EEG inherently contrasts many nonface versus many face stimuli to promote an invariant face‐selective neural representation from cortices associated with face processing (Hagen et al. 2020). In adults, face‐selectivity arises over bilateral occipital cortices with a right lateralization already from a single sequence presenting 50 different faces (Rossion et al. 2015). Using the same FPVS paradigm, de Heering and Rossion (2015) exposed 4‐to‐6‐month‐old infants to similar streams of complex natural images and demonstrated a right‐lateralized face‐selective response in infants as young as 4 months old. Crucially, the face‐selective response was absent when the same stimuli were phase‐scrambled. Thus, by disrupting the perception of the item but preserving the hue, luminance, contrast and spatial frequency, researchers have demonstrated genuine face selectivity of neural circuits both in adults and in infants 4 months and older (see also Gao et al. 2018; Or et al. 2019). The FT‐EEG visual paradigm presents the crucial advantage that it necessitates only few stimulation sequences to obtain a high signal‐to‐noise ratio, thus, the required testing duration is much shorter compared to other electrophysiological approaches (Hoehl 2016; Peykarjou 2022). These abovementioned FT‐EEG seminal face studies relied on the same stimulus set, which, despite using variable natural images, presented some systematic visual organization leading to confounding biases. In particular, the faces being presented centrally allowed a visible face pattern to emerge across the set average, thus confounding the face image statistics toward non‐face‐selective cues. To overcome this issue, another stimulus set (hereafter, “Classic set”), was built using more off‐centered faces of different genders and facial expressions, and off‐centered nonface objects (see Supporting Information S1: Figure S2 for the set averages). This Classic set has since then been extensively employed to elicit viewpoint‐invariant face‐selective responses, and has proven resourceful to tackle typical and atypical face perception using fMRI (Gao et al. 2018) or scalp EEG in adults (e.g., Quek et al. 2018; Rekow et al. 2020), children (e.g., Vettori et al. 2020), infants (e.g., Leleu et al. 2020) and patients with intracranial EEG (e.g., Hagen et al. 2020).

For example, using the Classic set, Leleu et al. (2020) replicated the face‐selective response previously observed in 4‐to‐6‐months old infants (de Heering and Rossion 2015) in a group of infants aged only 4 months. However, these face‐selective responses were rather weak in the baseline condition. Interestingly, face‐selective response considerably increased when visual stimulation was combined with the body odor of the infant's mother (Leleu et al. 2020), suggesting a higher capability of face‐selective processing than expected from the unimodal visual condition. More recently, two other FT‐EEG studies measured face‐selectivity in infants of the same age. Kiseleva et al. (2024) compared the Classic set with a simplified set where items had a canonical pose centered on a uniform gray background. They replicated the results of Leleu et al. (2020) for the Classic set (i.e., a small but significant face‐selective response which increased with the mother's odor) and reported a stronger response to their simplified set (Kiseleva et al. 2024). However, the simplified set presented only‐neutral faces without external features (i.e., hairline, neck), thus impoverishing the response's selectivity by adding confounded, non‐face‐selective, cues (e.g., a systematic pink oval shape, see Figure S1B). In another study, Yan et al. (2024) employed grayscale stimuli composed of the main items (rather centered, but varying in viewpoints) over a scrambled background to test for face‐selectivity in 3–4, 4–6, 6–8 and 12–15 month‐old infants. Despite longer stimulus presentation times than the abovementioned studies (i.e., 250 ms instead of 167 ms), face‐selective responses were found only in infants older than 4 months.

Important factors should be considered in visual stimulations in infant research, as infant vision is known for its protracted development over many years (e.g., for review see Braddick and Atkinson 2011). Visual acuity itself develops substantially in early infancy, with newborns typically resolving only 0.5–2 cycles per degree (cpd), increasing to 3–10 cpd by 6 months and to 5–15 cpd by 12 months (Neijzen et al. 2025). This gradual increase constrains the level of detail infants can perceive, making stimulus size and contrast critical factors for effective face processing. In fact, the Classic set used in infants, whereby the whole stimuli were presented at 24°–28° of visual angle, comprised color faces subtending on average 13° of visual angle (7°–17°, Kiseleva et al. 2024; Leleu et al. 2020). Faces were thus within the resolvable range for 4 months old infants. By contrast, Yan et al. (2024) relied on smaller greyscale stimuli (12° in total size), where faces themselves subtended 5° on average (2°–6°), i.e., closer to the visibility threshold of what 4‐month‐old infants are able to resolve. We thus suggest that notably too small image size might account for the absence of face‐selectivity in infants younger than 4 months in this latter study.

In sum, while face perception has been documented to undergo a protracted development over many years (e.g., Pascalis et al. 2011; Quinn et al. 2019), face categorization in face‐selective neural circuits seems to emerge at least from the age of 4 months. Yet it could be hypothesized that if visual constraints imposed by the Classic set (Kiseleva et al. 2024; Leleu et al. 2020) would be mitigated while maintaining a high within and between category generalization, face‐selective responses could be captured even earlier. The present study thus aimed to determine whether face‐selectivity can be detected in infants aged 4 months and younger, using an FT‐EEG stimulation involving a large number of stimuli. As a first step, we designed and validated a new stimulus set (“New set”) specifically tailored to young infant's visual capacities, that is, low acuity and contrast sensitivity (Braddick and Atkinson 2011). Moreover, the New set's natural stimuli background promoted figure‐ground segregation through natural depth‐induced blur, which supports object recognition learning by enhancing the sharpness of close items in the foreground (Bjorklund 1997). At the same time, the New set retained high high‐level visual complexity through broad within and across category variability and a large number of stimuli presented rapidly as every 167 ms (i.e., 6 Hz). Relying on a frequency‐tagging approach in EEG, we first validated the New set in adults (Experiment 1) before applying the New set to infants aged from 2 to 6 months (Experiment 2). Our FT‐EEG paradigm indexes a face presentation frequency by presenting faces at regular intervals (here, 1 Hz = 1/s) within a stream of non‐face stimuli at a fast rate (i.e., 6 Hz). As a result, two distinct neural responses can be extracted from the EEG spectrum: the general visual response at 6 Hz, representing the neural synchronization to the visual stream of stimulation (including processed common to both face and nonface stimuli) and the face‐selective neural activity at exactly 1 Hz, which inherently isolates activity elicited by the response to face stimuli. We hypothesized that in adults, the New set would elicit a robust 1‐Hz face‐selective response with a minimal contribution from low‐level confounds, which we assessed by presenting the scrambled version of the same stimuli (Experiment 1). We expected that the 6‐Hz general visual response would not vary across sets. In infants, we expected an overall enhancement of face‐selective response at 1 Hz with the better‐matched stimuli of the New set, without set difference for the general visual response, as for adults. We additionally explored whether the advantage of the New set varied with age within the 2–6 month age range.

## 2 Material and Methods

The research was approved by the Ethics committee of the University of Hamburg. The anonymized associated data are made permanently available: http://doi.org/10.25592/uhhfdm.18250.

## 3 General Methods

### 3.1 Construction of the New Set

As the frequency‐tagging approach measures a contrast response, the New set substitutes both face and nonface stimuli of the Classic set. Indeed, enhancing the face‐selective response is sensitive to a better recognition of nonface stimuli as being excluded from the face category.

In order to maintain the most common characteristics with the Classic set, we matched images from the same categories whenever possible or finding a close categorical match when it was not (e.g., dishwasher vs. washing machine). From the THINGS database (Hebart et al. 2019) which comprises 1854 object categories from squared photographs, we obtained 142/170 nonface stimuli. Some images meant as nonface stimuli were modified to remove silhouettes (e.g., swimming pool without swimmers). Missing categories of the nonface stimuli and all portraits of the face stimuli were completed from license‐free images from Pixabay.com (collected in June 2023) or the first author's personal collection. Overall, the main criteria to select the pictures were a low background noise (e.g., uniform or blurry background) and a high contrast between the foreground (i.e., item) and background. Whenever possible, we picked diverse locations (e.g., outdoor/indoor) and counterbalanced the luminance/hue of the foreground versus background to minimize category‐related biases.

### 3.2 Intact and Scrambled Visual Stimuli

**FIGURE 1:**
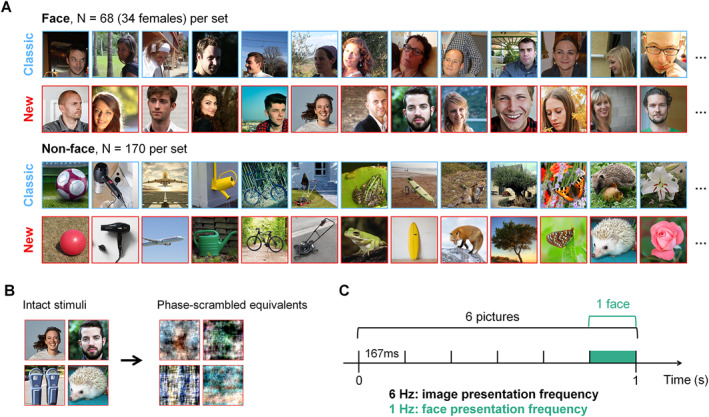
Stimuli and procedure. (A) Examples of stimuli from the Classic (blue) and the New (red) set, matched by category: each set included 170 non‐face stimuli and 68 faces used in both experiments. (B) During Experiment 1, the stimuli of both sets (here demonstrated for the New set) were available in their intact and phase‐scrambled versions, which were used in dedicated trials. (C) Example of one second of the FT‐EEG presentation paradigm, used in all trials of both experiments.

Stimuli from the Classic and the New sets consisted in color images of 400 × 400 px, each set containing 68 faces (34 females) and 170 nonface stimuli (Figure 1A). The face sets were each randomly split into 2 equivalent subsets (i.e., 17 females) such that 34 faces (50% of each sex) were to be presented in every trial. Trials either presented the stimuli in their original, “intact”, versions or after a phase‐scrambling manipulation (i.e., hereafter “scrambled”). The phase‐scrambling is obtained by disrupting the shape and structure of the stimuli (i.e., replacing the phase of each image by random coefficients; Rossion et al. 2015) while preserving the Fourier amplitude information, color, hue and luminance. In line with previous studies (de Heering and Rossion 2015; Gao et al. 2018; Or et al. 2019; Rossion et al. 2015), this scrambled condition helps to disentangle the low‐level contributions to the face‐selective response and controls for other confounding factors. Based on the aforementioned studies, it is expected that these dedicated trials do not yield a face‐selective response.

We conducted a full‐set and a cropped‐faces comparison of low‐level cues across sets to ensure their concordance (Figure S2). Overall, the New set appeared brighter than the Classic set for full images. The faces were slightly larger (+30 px on average) and overall brighter in the New set (Figure S3, see Supporting Information methods II and Table S1 for the full analyses).

### 3.3 Frequency‐Tagging Procedure

The FT‐EEG experiment was coded in Python from the template made available by Rekow (2024) and it was presented using Psychopy (Peirce et al. 2019). The stimulus presentation parameters were inspired from previous studies (Kiseleva et al. 2024; Leleu et al. 2020). Sequences lasted 35.666 s, starting with a fade‐in of 0.833s (0%–100% contrast), then 34s of full‐contrast stimulation ending with a fade‐out of ramping down contrast over 0.833s. Stimuli were displayed at a fast rate of 6 Hz, that is, each image lasted 167 ms on screen. Every second thus comprised six images, introducing one face as 6th stimulus to elicit a face‐selective response at the corresponding 1‐Hz frequency in the EEG spectrum. The paradigm allows to dissociate two distinct neural responses within a single stimulation, that is, the *general visual response* at 6 Hz (reflecting the common visual response across all stimuli) and the *face‐selective response* at 1 Hz which is known to capture neural activity selectively associated with face categorization (Gao et al. 2018; Hagen et al. 2020; Rossion et al. 2015).

The two following experiments were run in the same sound‐attenuated and dimly‐lit room. Stimuli were presented at the center of a 3840 by 1080 pixel‐wide screen using a 120‐Hz refresh rate (i.e., 20 frames/s).

### 3.4 Experiment 1: New Set Validation in Adults

#### 3.4.1 Participants

We recruited *N* = 19 healthy adults (10 females) aged 19 to 35 years‐old (mean: 25) from the University campus in exchange for course credit. All gave informed consent before starting the experiment. They declared normal or corrected‐to‐normal vision, and no history of psychiatric, neurological issues, or sensory impairment. Since, to our knowledge, no previous FT‐EEG study evaluated effects of Set in adults, we calibrated our sample size based on previous face categorization studies which relied on the Classic set (Quek et al. 2018; Quek and Rossion 2017; Retter et al. 2020; Retter and Rossion 2016). Their samples ranged from 13 to 20 participants, for an average of *N* = 16. We thus aimed at a minimum of *N* = 16 included participants and recruited three more volunteers before preprocessing the data, to account for data exclusion.

#### 3.4.2 Procedure

Participants had their head on a chinrest and were seated at a 1.55‐m distance from the screen (visual angle ≈ 9.2°). The FT‐EEG experiment consisted in 24 trials corresponding to 3 repetitions of one block of 8 trials (*Set* (Classic, New) × *Image* (intact, scrambled) × *Subset* (1, 2)) presented randomly within each block.

During trials, participants were asked to press the spacebar of the keyboard when they saw randomly appearing color targets (51 × 85 px) (i.e., orthogonal task: 4‒6 appearances per trial) to maintain their attention to the stimulation. Detection accuracy was high (mean ± SD: 99.7 ± 0.4%) and mean response time was 585 ± 65 ms confirming that participants paid continuous attention to the streams of stimuli. In addition, after the end of the FT‐EEG experiment and before debriefing, participants saw successively 4 series of 6 scrambled stimuli and were asked whether they perceived a face among the stimuli of each series. Stimuli were randomly picked from those used in the EEG experiment (i.e., 2 series for each set) and each series contained one face (this was not mentioned to the participant beforehand). The faces remained unfound over 99% of the time.

#### 3.4.3 EEG Acquisition and Preprocessing

EEG was continuously acquired at 1000 Hz from an EasyCap active montage of 57 channels (see Figure S4A) arranged according to the 10‒10 classification system and using AFz as passive ground and Cz as reference (i.e., 55 recording channels). The datasets were first bandpass filtered (Butterworth filter 0.1–100 Hz, 4th order) and down‐sampled to 200 Hz, before being cropped into 36‐s‐long segments starting from the onset of the sequence (i.e., including +333 ms after fade‐out).

Data signal correction was based on an Independent Component Analysis (ICA) using the Square algorithm to remove eye blinks and high amplitude artifacts (> 200 μV) over central and frontal channels (2‒5, mean ± SD: 3.32 ± 0.8). Remaining noisy channels were interpolated (*N* = 1 for 3 participants) and electrodes were re‐referenced to a custom average (excluding non‐symmetrical electrodes which would bias the strength of the signal in one direction, Figure S4A). Epochs were then segmented from 0.833s after the sequence started for a duration of 34 s (i.e., 34 exact face cycles, ending at the end of the fade‐out), and sorted by condition. After averaging the epochs by condition in the time domain, a fast Fourier transform (FFT) was performed with a resolution of one bin every ≈ 0.029 Hz. The amplitude of the signal was baseline corrected from adjacent noise, and noise was defined as the range of 20 neighboring bins (12 on each side, excluding the max/min and immediately adjacent values).

The following steps were done in parallel both for the 1‐Hz face‐selective and the 6‐Hz general visual responses. (1) To quantify how many harmonics were to be used in subsequent analyses, we computed the group average of each condition and averaged the 55 channels together. *Z*‐scores were computed on the amplitude spectra of this averaged channel as the difference between each frequency bin and the mean surrounding noise, divided by the standard deviation of the noise. We retained the highest number of consecutively significant harmonics across conditions (Retter et al. 2021). For the face‐selective response, it resulted in 15 harmonics (i.e., 1–15 Hz, excluding the 6th and 12th which overlap with the general response) and for the general visual response, the total of 8 harmonics (i.e., 6–48 Hz) was reached. Individual segments were then summed together to obtain a comprehensive quantification of each neural response (e.g., summing of the 8 chunks of the general visual response into one summed amplitude value representing the 8 harmonics). Future mentions of both responses (1‐Hz face‐selective response and 6‐Hz general visual response) will refer to the responses summed across their respective number of harmonics. (2) Next, on the summed responses, we defined regions of interest (ROIs) from the baseline‐corrected amplitude following a typical pipeline relying on the ranking of the highest amplitude electrodes and their contralateral corresponding electrodes. We thus defined the right (rOT: P8, PO8, P10, PO10) and left (lOT: P7, PO7, P9, PO9) occipital‐temporal regions and the middle occipital region (mO: PO3, PO4, O1, Oz, O2) for the face‐selective response (Or et al. 2019; Rossion et al. 2015). By applying a similar ranking procedure for the general visual response, a single ROI (mO) comprised PO4, PO3, PO8, O1, Oz, O2 represented the best 10% electrodes.

#### 3.4.4 Statistical Analyses

First, to quantify the differences across conditions, we ran separate analyses of variance (ANOVAs) for each visual response (i.e., 1 and 6 Hz, respectively), as the inherent difference in power across responses would overstep the differences across conditions. These ANOVAs were run on amplitude (μV), *F* values are reported with partial eta squares (*η*_*p*_^*2*^) as effect sizes and epsilon from Greenhouse‐Geiser correction when violation of sphericity was met. Student *t*‐tests were performed as post‐hoc tests to decompose significant interactions. We used *p* < 0.05 as significance threshold and *p*‐values are reported after an FDR correction for false‐discovery rate (Benjamini and Hochberg 1995) in case of multiple comparisons (*p*_*FDR*_). For the 6‐Hz general visual response, *Set* (Classic, New) and *Scrambling* (intact, scrambled) were within‐subject factors using the response recorded over mO; for the 1‐Hz face‐selective response *Set* (Classic, New), *Scrambling* (intact, scrambled) and *ROI* (rOT, lOT, mO) were the within‐subject factors. Bayesian statistics were used to complement absence of significance for effects in the ANOVAs which were relevant to our hypotheses, namely the absence of significant *Set* difference in (1) the Scrambled conditions at 1 Hz and (2) to the 6‐Hz general visual response. We thus conducted Bayesian ANOVAs using the *BayesFactor* package (Morey and Rouder 2023) in R to quantify evidence for the null hypothesis (from 10,000 iterations). These Bayes Factors are reported as BF_10_ and are interpreted according to Jeffrey's scale (Jeffreys 1991).

Second, *Z*‐scores (as defined above) were calculated at group level to estimate the group significance of the response in each condition for both the 1‐Hz face‐selective response and 6‐Hz general visual response. The number of individually significant *Z*‐scores for the face‐selective response were compared across *Set* using paired *t*‐tests (two‐tailed, *p* < 0.05).

Finally, for each Set separately, internal consistency estimates based on split‐half correlations were assessed for the face‐selective response using a Pearson correlation on the amplitude averaged across the 3 ROIs for odd versus even trials (FDR corrected for 2 tests). To assess whether the internal consistency differed between conditions, the correlation coefficients (R) were compared using Fisher's Z transformation for dependent correlations.

#### 3.4.5 Results of Experiment 1

**FIGURE 2:**
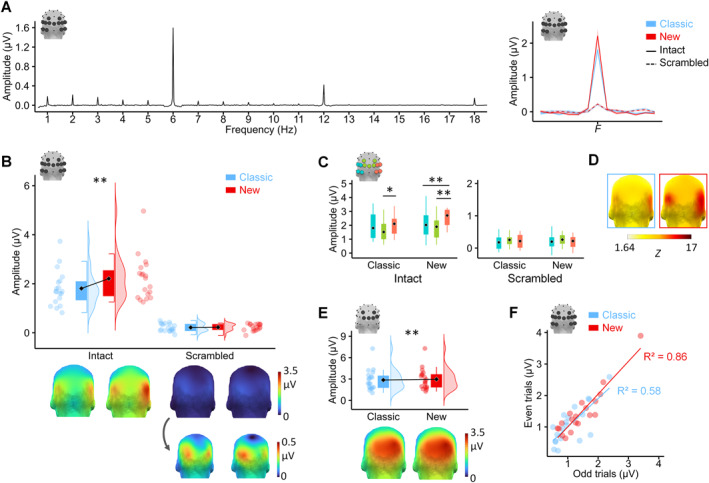
FT‐EEG results for Experiment 1. (A) FFT spectra representing the amplitude from the average of the electrodes of the ROIs of the face‐selective response. Left: amplitude obtained across all conditions and participants, distributed over the harmonics of the periodic presentation (i.e., 1 Hz or 6 Hz and integer multiples). Right: summed amplitude of the face‐selective response (1‐to‐15 Hz) to the Classic (blue) and New (red) sets and for the intact (solid line) and the scrambled condition (dashed line). Lines represent group mean and shaded areas represent SEM. (B) Amplitude of the 1‐Hz face‐selective response and harmonics (1‐to‐15 Hz) according to *Scrambling* by *Set* and (C) according to *ROI* (electrodes defining a ROI are marked in blue, green and red, see insert). 3D back‐view topographies display the scalp group mean amplitude (μV). (D) Topographical repartition of the response significance illustrated with *Z*‐scores for the intact condition. (E) Amplitude (μV) of the 6‐Hz general visual response (and harmonics [6‐to‐48 Hz]) over the ROI mO. 3D back‐view topographies display the scalp group mean amplitude (μV). (F) Split‐half correlations for internal consistency within sets. The amplitude in the intact condition was averaged over the 3 ROIs and correlated between odd and even trials. Black diamonds represent the mean and dots represent individual participant's data (*N* = 19) along with their distribution, * *p*_FDR_ < 0.05, ** *p*_FDR_ < 0.01.

The general visual response (6 Hz and harmonics until 48 Hz) yielded a main effect of *Set* (*F* (1, 18) = 7.44, *p* = 0.01, *η*_*p*_^*2*^ = 0.29); displaying a higher response for the New set (mean ± SEM: 2.96 ± 0.33 μV > 2.86 ± 0.33 μV, Figure 2E). This result was however not supported by the Bayes analyses which provided moderate evidence in favor a null effect for Set (BF_10_ = 0.32, error = 1.15%). Regarding the face‐selective response (1–15 Hz, without 6 and 12 Hz), we found main effects of *Scrambling* (*F* (1, 18) = 182.25, *p* = 0, *η*_*p*_^*2*^ = 0.86) and *Set* (F (1, 18) = 12.65, *p* = 0.002, *η*_*p*_^*2*^ = 0.41) and a *Scrambling* × *Set* interaction (*F* (1, 18) = 14.83, *p* = 0.001, *η*_*p*_^*2*^ = 0.45) whereby the New set (2.21 ± 0.2 μV) yielded higher responses than the Classic set for the intact condition (1.81 ± 0.17 μV, *t*_18_ = 3.66, *p*_FDR_ = 0.002), but no difference was found for the scrambled condition (0.23 ± 0.03 vs. 0.22 ± 0.04 μV, respectively, *t*_18_ = 0.39, *p*_FDR_ > 0.7; Figure 2B). The Bayesian analysis corroborated the absence of amplitude difference in the scrambled condition yielding moderate evidence in favor of the null hypothesis (BF_10_ = 0.21, error = 1.57%). In addition, the significant effects of *ROI* (*F* (2, 36) = 5.59, *p* = 0.008, *η*_*p*_^*2*^ = 0.24), *Scrambling* × *ROI* (*F* (2, 36) = 8.38, *p* = 0.001, *η*_*p*_^*2*^ = 0.32) and *Scrambling* × *Set* × *ROI* (*F* (2, 36) = 3.31, *p* = 0.05, *η*_*p*_^*2*^ = 0.16) implied that amplitudes were different in the intact condition across ROIs: there was a right hemispheric dominance in the intact condition which in turn was especially pronounced for the New Set (rOT > lOT: 2.71 ± 0.26 > 2.02 ± 0.23 μV and rOT > mO: 1.89 ± 0.21 μV; *t*s > 2.35, *p*_FDR_ < 0.03, Figure 2C). In contrast, post‐hoc *t*‐tests in the scrambled condition found no significant differences across ROIs (*t*s < 1.27, *ps*_FDR_ > 0.22), and Bayesian analysis provided moderate evidence for similarity of amplitude across ROIs in the Scrambled condition (BF_10_ = 0.23, error = 1.17%). The full extent of these two ANOVAs are available in Tables S2 and S3 (Supplementary results I).

Group level Z‐scores indicated that the face‐selective response was only significant for intact sequences (*Z*s > 12, *p*_*FDR*_s < 0.0001; with *Z*s < 1.38, *p*_*FDR*_s > 0.11 for scrambled sequences). With closer inspection, group *Z*‐scores demonstrated a high significance over all ROIs (*Z* range: 21–34) for both Sets in the Intact condition (Figure 2D). At the individual level, about 96% of the 13 electrodes of the ROIs reached significance (the number of significant electrodes per individual did not significantly differ across Set, *t*_*18*_ = 0.29, *p* = 0.77), but across the 55 scalp electrodes, the New Set yielded a higher number of individually significant electrodes (i.e., 37.7 ± 10 > 32.4 ± 11 for the Classic Set, *t*_*18*_ = 3.7, *p* = 0.002).

Finally, split‐half correlations were highly significant (*R* ^2^ > 0.58, *p*_*FDR*_ < 0.001, Figure 2F) indicating strong within‐condition consistency for both stimulus sets. The correlation strengths did not significantly differ between sets (*Z*_*fisher*_ = 1.82, *p* = 0.07) suggesting that the internal consistency of the face‐selective responses were indistinguishable between sets.

#### 3.4.6 Discussion

In Experiment 1, we found a stronger face‐selective response for the New set than for the Classic set (i.e., +22% of increase) while at the same time, the responses did not appear to differ for the scrambled images. Thus, both stimulus sets do not differ in their low‐level contributions. Behavioral data (see *Procedure*) additionally indicated that participants were unable to explicitly detect scrambled faces among nonface scrambled stimuli. The stronger face‐selective response in the New set was characterized by a right hemispheric dominance which confirms that the high‐level face‐selective responses (Rossion and Lochy 2021) were driving the difference across sets. In addition, the split‐half correlations to estimate internal consistency within both sets were very high for both the Classic and the New sets and they did not differ between sets. This suggest that both sets estimated the same visual function. The weakly higher 6‐Hz general visual response to the New set was likely driven by the inherent overlap between the 6th harmonic of 1‐Hz face‐selective response (i.e., 6 Hz) and the first harmonic of the general visual response (i.e., 6 Hz; see Supplementary results II for supporting evidence). While this effect was not supported by Bayesian analyses (favoring a null effect for *Set*), our conclusion would remain cautious. Our design was not specifically tailored to test this effect, which, if replicated, constitutes a limitation that future studies could address. Altogether, the results from Experiment 1 demonstrated that the New set produced a valid measure of face‐selectivity in adults, allowing us to consider its implementation in an infant population.

### 3.5 Experiment 2: Face‐selectivity in infants

#### 3.5.1 Participants

We recruited 53 infants, of whom *N* = 1 was excluded due to technical issues during recording and *N* = 5 did not obtain enough data after epoch selection, leading to a final sample of *N* = 46 infants (57–200 days, mean: 131, *N* = 26 females). All were healthy at the time of testing and no parent reported a history of sensory or neurological issues. Informed consent was obtained by a legal guardian before testing. We considered two age groups by dividing the sample into 2 equal halves using the median age value (149 days old, obtained by 3 infants). The younger half (*N* = 23, 14 females) included infants from 57 to 149 days old (100 days on average) and the older half (*N* = 23, 12 females) included infants aged from 149 to 200 days old (162 days on average).

Sample size was estimated a priori from Kiseleva et al. (2024), whose study compare differences between sets for face categorization in 4‐month old infants, thus close to our comparison between the Classic and the New set. Using G*Power 3.1.9.7 (Faul et al. 2007), we found an effect size of *f* = 0.639 based on their effect of the between‐subject factor stimulus Set (F(1, 39) = 16.2, *p* < 0.001, ηp^2^ = 0.29). According to G*Power, for a repeated‐measures ANOVA (within‐subjects factor) with a significance level *α* = 0.05 (two‐tailed) and a power 1−*β* = 0.95, this yields a minimal recommended sample size of *N* = 12. However, since we aimed to include infants of a younger age, we continued recruitment beyond that to increase statistical power and account for expected exclusions due to infant EEG attrition. More participants were included as recruitment was open for a period of 3.5 months (January 4 to April 15, 2024).

#### 3.5.2 Procedure

Stimuli were the same as in Experiment 1 (Figure 1), but only the Intact versions were used in Experiment 2.

Infants were seated in a high chair or on their parent's lap at a 60‐cm viewing distance from the screen (visual angle ≈ 23.5°). Trials of the intact condition (counterbalancing the *Set* [Classic, New]) were launched manually by the experimenter when the infant, who was monitored by a webcam, was looking toward the screen. Short toy sounds (e.g., “boing”) coming from behind the center of the screen were manually launched to reorient the infant's gaze when necessary. The sounds sporadic occurrences did not interfere with the FT‐EEG recording. Testing was stopped when infants manifested tiredness or disinterest, or at parental request. We recorded on average 7.65 trials per infants (range: 4–12) and visual attention was assessed trial‐by‐trial before inclusion ((Peykarjou 2022); see below *EEG acquisition and preprocessing*). The number of retained sequences did not differ between conditions (average of 3.57 ± 1.34 [range: 2–8] for the Classic vs. 3.70 ± 1.21 (range: 2–6) for the New set; *t*_*45*_ = 0.8, *p* = 0.43).

#### 3.5.3 EEG Acquisition and Preprocessing

EEG acquisition parameters and first preprocessing steps (filtering, downsampling and cropping) were common to that of Experiment 1, with the exception that infant's EEG was acquired from a custom 34‐channel montage (32 recording electrodes following the 10–10 classification, see Figure S4B). Since the ICA is usually not adequate to isolate typical artifacts in infant EEG, we applied the *Artifact Blocking* algorithm (Fujioka et al. 2011; Mourad et al. 2007) with a threshold of ± 500 μV on 36‐s‐long segments obtained from cropping starting from the onset of the trial (including +333 ms after fade‐out). Next, we interpolated pre‐identified noisy channels (*N* = 1 for four infants). Electrodes were next re‐referenced to a custom average (see Figure S4B) and epochs were segmented from 0.833 s after the trial start for a duration of 34 s (i.e., 34 exact 1‐s face cycles) meaning that the frequency resolution was ≈ 0.029 Hz, as for Experiment 1. Furthermore, epochs were screened for exclusion by assessing the significance of the 6‐Hz general response on individual epochs as is customary done in infant FT‐EEG studies (e.g., Leleu et al. 2020; Peykarjou 2022). We kept epochs with at least one *Z*‐score > 2.32 (*p* < 0.01, one‐tailed) over POz, O1, Oz or O2 at six or 12 Hz. This step resulted in the exclusion of 18/352 trials (i.e., 5.11%, 0.4 trial on average per participant). The remaining clean segments were sorted according to condition and were averaged in the time domain before calculating the FFT. Noise was defined as the 30 neighboring bins (16 on each side, excluding the max/min and immediately adjacent values), using the closer range of 1st to 5th bin for linear detrending and the further range of 6^th^ to 16^th^ for *Z*‐score calculation.

The harmonic selection followed the same procedure as for the adult EEG in Experiment 1. Group Z‐scores (*Z* > 1.64, *p* < 0.05, one‐tailed) computed on the average of all electrodes yielded 4 harmonics for the face‐selective response (1–4 Hz) and 8 harmonics for the general visual response (6–48 Hz). The following analyses considered the sums of respective harmonics, and as in Experiment 1, we refer to these sums as “response”. Considering that infant's signal‐to‐noise ratio is known to be lower and inter‐individual anatomical variance higher, we divided the 17 channels posterior to the midline into 3 ROIs for the face‐selective response: rOT (CP6, P4, P8, PO8, P10), lOT (CP5, P3, P7, PO7, P9) and mO (O1, O2, PO3, PO4, Oz, POz, Pz). Since we analyzed responses from a large age range, including infants younger than tested before, we preferred to rely on this more exploratory approach to avoid missing out age‐dependent effects. For the general visual response, the typical single middle occipital ROI mO comprised POz, O1, Oz and O2 (Peykarjou 2022).

#### 3.5.4 Statistical Analyses

As for Experiment 1, the analyses of variance were separately run on the EEG amplitude (μV) of both visual responses (1‐Hz face selective and 6‐Hz general visual responses). The following factors were used: for the 6‐Hz general visual response, *Set* (Classic, New) was the within‐subject factors and *Age* (below vs. above the median value) was the between‐subject factor. For the 1‐Hz face‐selective response *Set* (Classic, New) and *ROI* (rOT, lOT, mO) were the within‐subject factors and *Age* (below vs. above the median value) was the between‐subject factor. As for Experiment 1, Bayesian analyses were performed in cases non‐significant effects for the 6‐Hz general visual response were found in the ANOVA (*p* > 0.05). Bayes factors (BF_10_) are reported to quantify evidence in favor of the null hypothesis. *Z*‐scores were calculated at group level to estimate the group significance of the response in each condition and frequency. To compare the number of individually significant *Z*‐scores for the face‐selective response across *Set* we used paired *T*‐tests (two‐tailed, *p* < 0.05).

#### 3.5.5 Results of Experiment 2

**FIGURE 3:**
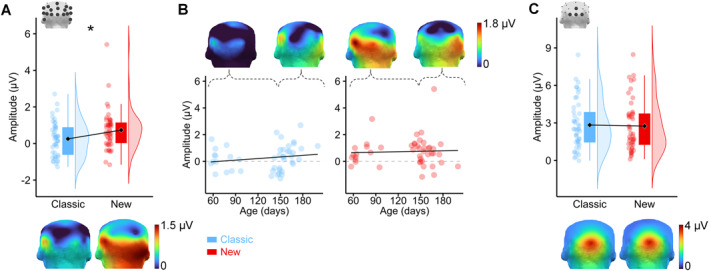
FT‐EEG results for Experiment 2. Amplitude of (A) the face‐selective response (1 Hz and harmonics, 1–4 Hz) according to the main effect of *Set*, (B) for the infants below and over the median age and (C) for the general visual response (6 Hz and its harmonics until 48 Hz). Black diamonds represent the mean and dots represent individual data (*N* = 46) along with their distribution, * *p* < 0.05.

The face‐selective response reached a higher amplitude (*F*(1, 44) = 6.97, *p* = 0.01, *η*_*p*_^*2*^ = 0.14) for the New set (mean ± SEM: 0.73 ± 0.16 μV) compared to the Classic set (0.25 ± 0.14 μV, i.e., increase by almost 3 times, Figure 3A). This effect was qualified by an *Age* × *Set* interaction which suggested that the youngest infants (*N* = 23, mean age: 100 days) benefitted more from the New set (+0.85 ± 0.25 μV) than the older half of the sample (*N* = 23, mean age: 162 days; Figure 3B; +0.11 ± 0.27 μV). Conversely, there was no significant difference in amplitude across *Set* for the general visual response (*F*(1, 44) = 0.02, *p* = 0.89, *η*_*p*_^*2*^ < 0.001). Bayes analyses provided moderate evidence for a similar amplitude across sets for the general visual response (BF_10_ = 0.22, error = 0.02%; mean ± SEM: 2.79 ± 0.28 μV, Figure 3C). The outcomes of the ANOVAs are available in Tables S5 and S6.

For the New set over the range of 4 harmonics, the response across the whole group (*N* = 46) was significant for all ROIs (*Z*: 3.08–3.62, *p*s < 0.001) and the average of all channels (*Z* = 5.10) while no corresponding *Z*‐score reached significance for the Classic set (0.32–1.20, *p*s > 0.11; Table S7) on the sum of 4 harmonics. A significant group response was nonetheless measured for the Classic set over a typical face‐responsive electrode (i.e., P7: *Z* = 2.45) for the 1^st^ harmonic. Regarding individual *Z*‐scores, more infants obtained significant responses over individual posterior channels in the New set (6.88 ± 1.96 infants (mean ± SD)) compared to the Classic set (5.88 ± 2.57; *t*_16_ = 2.24, *p* = 0.04).

**FIGURE 4:**
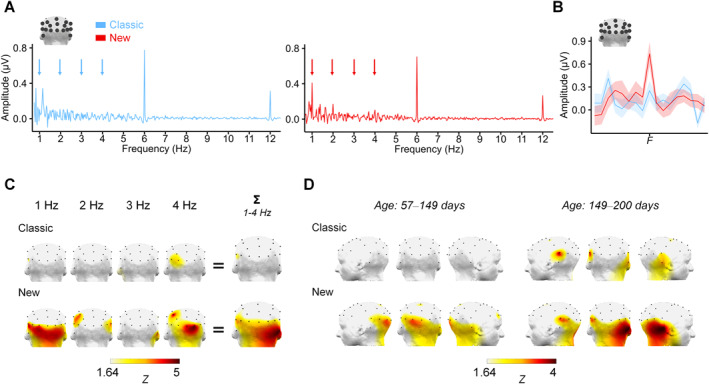
Frequency spectra and harmonic significance of the face‐selective response assessed by Z‐scores in infants. (A) FFT spectra (μV, baseline corrected) in response to the Classic (left, blue) and the New (right, red) sets, averaged over all posterior electrodes. Arrows indicate the first 4 harmonics of the face‐selective response. (B) Face‐selective response amplitude summed across the 4 significant harmonics. The plain lines represent the group average (*N* = 46, blue: Classic set, red: New set) and the shaded areas represent the SEM. (C) 3D back‐view topographies of the group *Z*‐scores for each harmonic and for the sum of the 4 harmonics (right) of the face‐selective response as a function of *Set*. (D) 3D back‐view topographies of the *Z*‐scores of the face‐selective response (summed for 4 harmonics) for the infants below and above the median age (*N* = 23, respectively) for each set of stimuli.

Most impressively, the face‐selective response was virtually absent in the Classic set for the younger half of the sample, as no *Z*‐score reached significance (i.e., scalp‐wide, among the 17 posterior electrodes separately or averaged together, or over the 3 ROIs). In addition, the responses to the New set in the same age group demonstrated that 53% of the 17 electrodes became significant (1.65 < *Z* < 3.53), as well as the 3 ROIs (*Z* range: 2.12–3.56, Figure 4). For the older half of the sample, the face‐selective response was notably well established with the Classic set (i.e., 24% of the 17 electrodes, 2 of 3 ROIs and the average of the posterior electrodes obtained *Zs* > 1.64, *ps* < 0.05). Still, as with adults (see Experiment 1), older infants displayed a more robust face selectivity with the New set, with 35% of the posterior electrodes reaching significance (*Z* range: 1.71–3.72, Figure 4B). Interestingly, while the left and right ROIs were significant in all conditions except for the younger half of infants with the Classic set (which had no significant response altogether), the middle occipital ROI was only significant with the New set in the younger half of the sample (*Z* = 2.97) but not for the older half of the sample (see Tables S7 and S8 for the complete series of *Z*‐scores).

## 4 Discussion

While neuroimaging studies suggested face‐selectivity in infants as young as 2 months (Kosakowski et al. 2024), frequency‐tagging EEG studies relying on a faster presentation rate of stimuli reported a later onset from around 4 months (Yan et al. 2024). Here we hypothesized that the onset of neural face selectivity in infancy might have been underestimated due to the stimuli's visual complexity. We designed a New stimulus set to enhance rapid face categorization with the least compromise on high‐level face‐selectivity, that is, while maintaining the highest within‐ and between‐category variability. The rationale behind the generation of the New set was to gather existing natural images and to match the semantic content, that is, the visual categories, proposed by the Classic set which has been used in numerous previous studies. The New set was created such that the stimuli presented a higher visibility of items, by selecting larger item size and image brightness associated with a smoother background. These manipulations were thought to ensure the measure of face‐selectivity in infants who exhibit a relative low visual acuity. Using a FT‐EEG paradigm we compared face‐selectivity yielded by the New set compared to the Classic set both in adults and infants aged 2–6 months. In adults, the 1‐Hz face‐selective response had a stronger amplitude for the New set than for the Classic set. Most importantly, the younger half of our infant sample (mean age = 100 days) featured a face‐selective response which was not detected with the Classic set. Therefore, we concluded that previous studies may have underestimated the face processing skills of young infants.

The stronger face‐selective response with the New set in adults suggests easier categorization of faces. Linking explicit behavioral face detection and FT‐EEG measures, the amplitude increase of the face‐selective response has previously been linked to higher face visibility (Liu‐Shuang et al. 2022; Quek et al. 2018) and the actual number of faces that one is able to detect (Retter et al. 2020). In the case of the New set, this might partially stem from a higher homogeneity of the face stimuli and/or of their higher dissimilarity with the other nonface stimuli, presumably arising from our selection parameters (notably more homogeneous background and more vivid colors). Since this FT‐EEG approach measures a contrast response between faces and non‐face stimuli, the stronger amplitude very likely relates to a more pronounced divergence between the faces and nonface objects (Rossion et al. 2015). Most crucially, the New set strengthened face‐selective indices as a higher right‐hemispheric dominance was measured as well. The right lateralization of the neural responses to faces is a well‐documented characteristic of face‐selectivity (e.g., De Renzi et al. 1994; Duchaine and Yovel 2015; Jonas et al. 2016). The present results thus suggest that the face stimuli of the New set activated the face network more often than those of the Classic set. Importantly, the amplitude increase over the right hemisphere was not associated with an increase over the middle‐occipital area. Thus, it seems unlikely that lower‐level cues such as face‐specific spatial frequency or contrast, typically associated with neural activity of the primary visual cortex (Boynton et al. 1999), caused higher face‐selectivity. The responses to the scrambled conditions of both sets were indistinguishable, corroborating the idea that the stimuli of the New set are not associated with more low‐level confounds than stimuli of the well‐validated Classic set (Or et al. 2019; Rossion et al. 2015; see Gao et al. 2018 for evidence in fMRI). Additional analyses confirmed that both sets did not differ in their inherent internal consistency for assessing face‐selective neural activity. Finally, both the group and the individual significance of the response (calculated through *Z*‐scores) were stronger for the New set, suggesting a high reliability in the New set. Altogether, these findings from Experiment 1 established that the New set offers a valid face‐selectivity measure, which appears even stronger than for the Classic set in the mature adult brain.

Impressively, we show that the youngest infants of our sample were able to categorize variable faces when quickly displayed among nonface objects and immediately backward masked by the next stimulus. Actually, at least from 3 months of age, infants possess some color consistency (Skelton et al. 2022), viewpoint invariance in shape perception (Kraebel et al. 2007), bases of contour processing (Gerhardstein et al. 2004) and object unity (Johnson and Aslin 1995), that is, fundamental bases for complex invariant visual object recognition. Our results are in stark contrast to those of a recent similar FT‐EEG study on category‐selectivity which did not find evidence for face‐selectivity in infants younger than 4 months old (Yan et al. 2024). Yan et al. (2024) used grayscale images over a scrambled background, which may have overstrained younger infant's figure‐ground segregation ability. By contrast, employing fMRI, Kosakowski et al. (2024) reported evidence of early face selectivity from 2 months onward. The latter study actually employed color videos of faces over a black background: in addition to the easy figure‐ground segregation allowed by the dark background, the dynamics brought by the short video clips likely helped infants recognizing the visual input (Kellman and Short 1987). Indeed, evidence has suggested that when infants can access associated meaningful cues, their face(like)‐selective neural response increases, as for example observed when simplifying the stimuli (Kiseleva et al. 2024) or by adding maternal body odor at 4 months (Leleu et al. 2020; Rekow et al. 2021). Thus, alongside the neuroimaging data (Kosakowski et al. 2024), we expected that FT‐EEG face‐selectivity could be measured earlier than 4 months of age even with static faces, a larger stimulus sets than used in fMRI designs, and similar stimulus presentation rate as many previous FT‐EEG studies in infants from 4 months of age (de Heering and Rossion 2015; Kiseleva et al. 2024; Peykarjou et al. 2024; Rekow et al. 2021, 2024). Contrary to Yan et al. (2024) who employed a slower presentation rate (i.e., 4 Hz = 4 images/s) and to Kosakowski et al. (2024) who presented 2.7‐s‐long video clips as stimuli, we demonstrated here that longer presentation times were not *necessary* to promote face‐selectivity. By enhancing the stimuli' saliency (while maintaining natural and highly variable images as opposed to e.g., Kiseleva et al. (2024)), our stimuli may have better aligned with infant's natural face exposure experience. Infant‐perspective data from head‐mounted cameras actually estimated that infants of 1‐to‐3 months experience most interactions with faces distant of around 70 cm (Jayaraman et al. 2015) and 60% of those last for 5 s or less (Jayaraman and Smith 2019). These face exposure statistics certainly support the development of the face‐selective network to respond to real‐life challenges.

The New set was built with the purpose of enhancing visual perception in typically‐sighted infants, and our age‐dependent analyses suggested that this was successfully achieved. Although adults gained from the New set too, their benefit was lower than one quarter increase (22%), whereas infant's amplitude increased by almost three times (189%). Importantly, considering the New set may better match some “optimal complexity” favored by infants (Kidd et al. 2012), we did not observe any impact on the general visual response (6‐Hz response) as the amplitude of the latter did not differ across conditions. The general visual response has been taken in infants FT‐EEG as a marker for visual attention (Peykarjou 2022), as the general visual response only arises when infants watch the sequence of periodically appearing stimuli for a sufficient amount of cycles so that the neural activity from the visual cortex synchronizes with the frequency of stimulation (i.e., the visual input changing at 6 Hz, i.e., every 167 ms). Moreover, we obtained an equivalent number of epochs per condition and infant, suggesting indistinguishable interest in the stimulation across infants. Thus, unspecific effects such as differences in overall visual attention cannot account for the gain in infant's face selectivity measured in response to the stimuli of the New set. Hence, it appears reasonable to infer that the measure of infant's face‐selective responses rather emerged thanks to the properties of the New set. This is notably demonstrated by the measure of response significance, since the younger half of the infants (100 days old in average) presented face‐selective activations for 53% of the analyzed electrodes in the New set, but for no electrode in response to the Classic set.

As we did not test here the scramble condition in infants, our conclusions are limited to the set difference for intact images in infants. However, since previous intact versus scrambled comparisons had reported similar response patterns across infants (de Heering and Rossion 2015) and adults (Gao et al. 2018; Or et al. 2019; Rossion et al. 2015), it suggests that the sensitivity to low‐level cues captured by the scrambling manipulation is comparable across ages, and that the neural face‐selectivity is truly enhanced by the visual parameters of the New set. By manipulating the item visibility, we aimed at increasing the chance for each individual face to be detected as such, so that fewer faces would likely fall below the visibility threshold of young infants. As evoked above, the neural response in FT‐EEG face categorization paradigms emerges only if a contrast is reliably measured throughout the sequence, as here between face and nonface items (Retter et al. 2020). Therefore, if many faces fall below the perceptual threshold of infant vision, the response may not emerge. We performed inter‐set correlations in both experiments to assess whether general and face‐selective responses correlated across sets (see Supplementary results IV for full details). In adults, the inter‐set correlation was significant for both the general and the face‐selective responses, and did not differ from the within‐set split‐half correlations, indicating that adult face‐selective responses were largely stable and set‐independent. In infants, by contrast, the inter‐set correlation was significant only for the general visual response, but not for the face‐selective response. This supports the idea that the New set enabled face‐selective responses in infants who did not show them with the Classic set, likely due to its better fit with their emerging face‐selective activity. One limitation of this complementary analysis, however, is the lack of significant response to the Classic set for one half of the sample. In this context, it should not be forgotten that with age, visual experience and exposure to more variable inputs, face categorization will comprise wider variations of faces (Rekow et al. 2024; Yan et al. 2024). Infants' face representation before 4 months may not yet generalize across smaller, darker, or more complex visual variations such as those in the Classic (e.g., Leleu et al. 2020) or other (Yan et al. 2024) sets. It is thus possible, that younger infants were able to detect faces within the Classic set too, but that their immature face‐selective regions responded similarly to all visual categories rather than selectively to faces. One may argue that the lack of early face‐selectivity observed for the Classic set was predominantly due to lower visual acuity of younger infants which was compensated by the New set. We consider this as a reasonable hypothesis. Thus, our study was important to demonstrate that such other factors prevented the observation of a face selective response in previous studies. Future studies simulating reduced visual acuity, for instance in adults, could complement this hypothesis. As a result, set selection for future studies should be made while aware of these set differences.

Finally, we observed that the face‐selective response of infants with the New set was more broadly distributed over the occipital scalp than the typical right‐dominant bilateral occipital temporal distribution in adults. In his *Interactive specialization* model of face perception development, M. H. Johnson (2011) ventured that the fine‐tuning of neural systems is accompanied by localization changes whereby a dedicated network would refine with increasing neural specialization. We thus propose that although early face‐selectivity may already activate typical face processing areas (Deen et al. 2017; Kosakowski et al. 2024), it could initially solicitate larger portions of the visual cortex (e.g., the middle occipital area), before refining to more specific occipital temporal cortices.

In conclusion, in line with the most recent brain imaging results, we provide evidence for face‐selective neural circuits in infants younger than 4 months old, capable of categorizing faces under tight presentation constraints. This efficient face processing most likely scaffolds the concurrent development of higher order face processing skills and paves the way for the emergence of higher order visual and social neurocognitive development.

## Author Contributions

**Diane Rekow:** conceptualization, data curation, formal analysis, funding acquisition, investigation, project administration, software, visualization, writing – original draft, writing – review and editing. **Tanisha Arya:** investigation, software, visualization, writing – review and editing. **Duygu H. Bayir:** investigation, writing – review and editing. **Brigitte Roder:** conceptualization, funding acquisition, project administration, writing – review and editing.

## Ethics Statement

The research was approved by the local ethics committee of the University on Hamburg (#2023_036).

## Conflicts of Interest

The authors declare no conflicts of interest.

## Supporting information


Supporting Information S1

## Data Availability

The anonymized preprocessed EEG data of both experiments are publicly available: http://doi.org/10.25592/uhhfdm.18250. The stimuli of the New set are available upon request to the corresponding author.
