# Supplementary material for: Emergence of Neural Face Selectivity in Infants Younger Than 4 Months Old
Source: Infancy. 2026 Feb 21;31(1):e70076. doi: 10.1111/infa.70076 (PMC12924696; doi:10.1111/infa.70076)
Supplement: Supplementary file 1 — Supporting Information S1 [file INFA-31-0-s001.pdf]

Emergence of neural face selectivity in infants younger than 4 months old

Diane Rekow, Tanisha Arya, Duygu H. Bayir, Brigitte Röder

Supplementary method I

By computing the average image of the set exemplars, it highlights obvious biases present in the stimulus set. For instance, from FigS1 A, B or C a discernable "face pattern" is seen, indicating that the individual exemplars of the respective sets overlap sufficiently in their image organization to provide a high consistency which might trigger non-selective categorization.

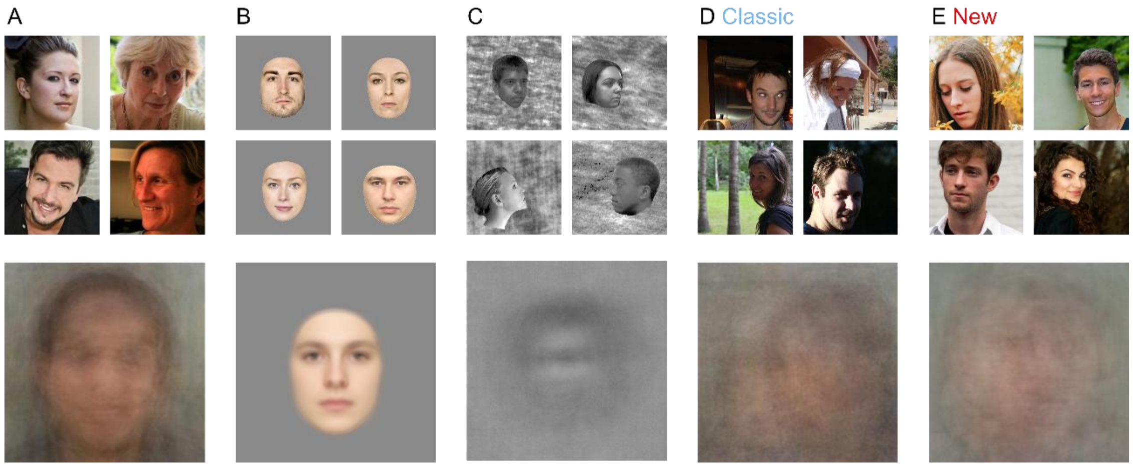

Figure S1. Examples of the reviewed stimulus sets. Examples of 4 stimuli are given for each set (top) and the whole face set average picture (bottom) from **(A)** the N = 48 stimuli from de Heering & Rossion (2015); **(B)** the N = 72 stimuli from Kiseleva et al. (2024); **(C)** the N = 144 stimuli from Yan et al. (2024); **(D)** the N = 68 stimuli from Leleu et al. (2020) which correspond to the Classic set used here and **(E)** the N = 68 stimuli from the New set of the present study. *See main manuscript for full references.*

Supplementary method II – Physical comparison across sets

Full set comparison across the Classic and the New sets

The Classic and New sets share important characteristics, some of which are a priori controlled (matched visual categories and variable viewpoints of multiple exemplars). The average image of the face and nonface stimuli subsets looked quite similar, although due to the more canonical positions in nonface item selection, a central shape was more discernable on the nonface average image of the New set (Figure S2). Objective measures were derived to compare the image statistics of both sets by calculating Student T-tests ($t_{df,}$, p > .05) and p-values are reported after an FDR correction for false-discovery rate (Benjamini & Hochberg, 1995) in case of multiple comparisons (p_{FDR}). The FDR correction is calculated by multiplying the p-value by the number total of tests divided by the rank of the given p-value across all comparisons.

First, to assess the overall contrast across stimuli, we computed the variance across the pixel values of each image for each set. No difference was revealed across the face vs. nonface stimuli within each set, nor across sets ($ts < 2$; $p_{FDR} > .19$). When the variance was analyzed within each of the RGB layer separately, no difference was found across sets for both subsets (nonface Classic vs. New and face Classic vs. New: $ts < 1.19$; $p_{FDR} > .47$). In addition, when comparing the face and nonface subsets within each set, there was no difference in the Green and Blue layers ($ts < 2.47$, $p_{FDR} > .06$). Both for the Classic ($t_{236} = 3.36$, $p_{FDR} = .006$) and the New set

(t_{236} = 3.84, p_{FDR} = .002), the face subsets presented a higher contrast in the Red layer than their respective nonface subset. Next, we compared the mean luminance (ranging from 0: dark to 1: bright) across pixels for each image. Interestingly, we obtained an overall more homogeneous luminance for the New set as there was no difference between the face and nonface stimuli (0.50 ± 0.13 vs 0.51 ± 0.12, respectively; t_{236} = 0.49, p_{FDR} = .63). By contrast, faces (0.43 ± 0.12) were darker than the nonface stimuli (0.47 ± 0.12, t_{236} = 2.63, p_{FDR} = .01) for the Classic set. In addition, the New set was brighter than the Classic set for both subsets (ts > 2.81, p_{FDR} < .01). These results are presented below in Figure S2. Although our measures suggest that the Classic set may have been less controlled, it remains possible that previous studies using this set accounted for such variability by adjusting luminance prior to stimulus presentation.

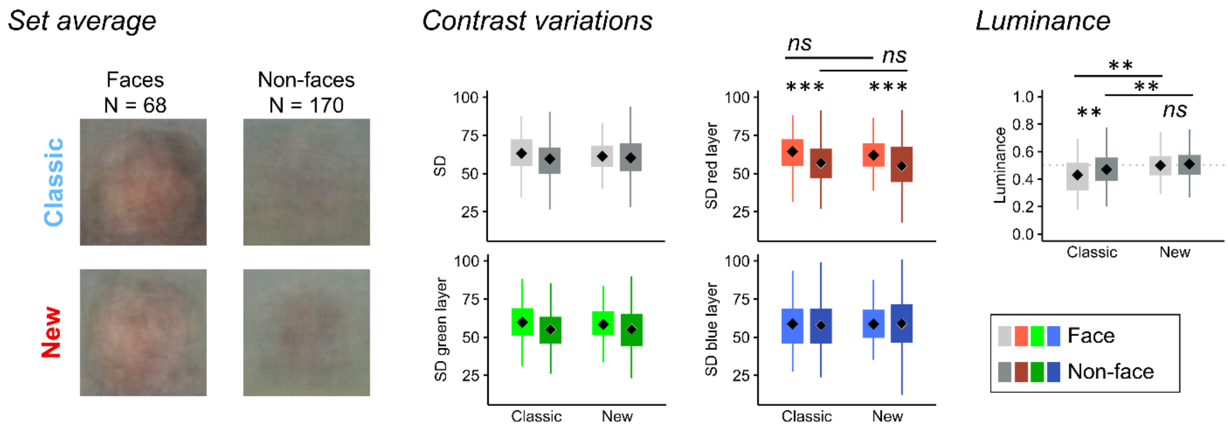

Figure S2. **Analyses of the image statistics across the Classic and the New sets and subsets (face vs. non-face stimuli) for the full images.** Black diamonds represent the mean. ns: p_{FDR} > .05; **: p_{FDR} ≤ .01; ***: p_{FDR} < .001.

Comparison of the cropped faces

Since in both the Classic and the New sets, faces covered a variable surface within each image, we extracted the face size information and adjusted the luminance calculation to this area to gain accuracy in the comparison across sets and disentangle the contribution of the image background. To that end, we used the *vision.CascadeObjectDetector* from Matlab 2024 which relies on the Viola-Jones algorithm (Viola & Jones, 2001). We combined 3 models as Face detectors: the FrontalFaceCart, FrontalFaceLBP and ProfileFace, to optimize the chances to detect the faces in our stimuli (MaxSize = 400 px, MergeThreshold = 4 (default)). Overall, even when combining the 3 models, some faces remained undetected (N = 20/68 for the Classic set and N = 10/68 for the New set), marking a significant difference across both sets as measured with paired T-tests (t_{66} = 2.11, p_{FDR} = .05). There was no difference for the ProfileFace model (p_{FDR} = .21) but the two remaining models performed better for the New set (FrontalFaceCart: N = 54 > N = 32/68, t_{66} = 4.26, p_{FDR} = .0003; FrontalFaceLBP: N = 47 > N = 31/68, t_{66} = 2.88, p_{FDR} = .01). The detected faces were automatically cropped to isolate them from the background, and the undetected faces were cropped manually.

Analyses of the face size showed that the New set displayed faces of ≈ 30px larger on average within the 400 x 400 pixels images (195 ± 41 vs 223 ± 44 px; t_{66} = 4, p < .001). In line with the previous luminance analysis, we confirmed that the faces of the New set were indeed brighter (Figure S3).

A summary of the results is presented in Table S1 below.

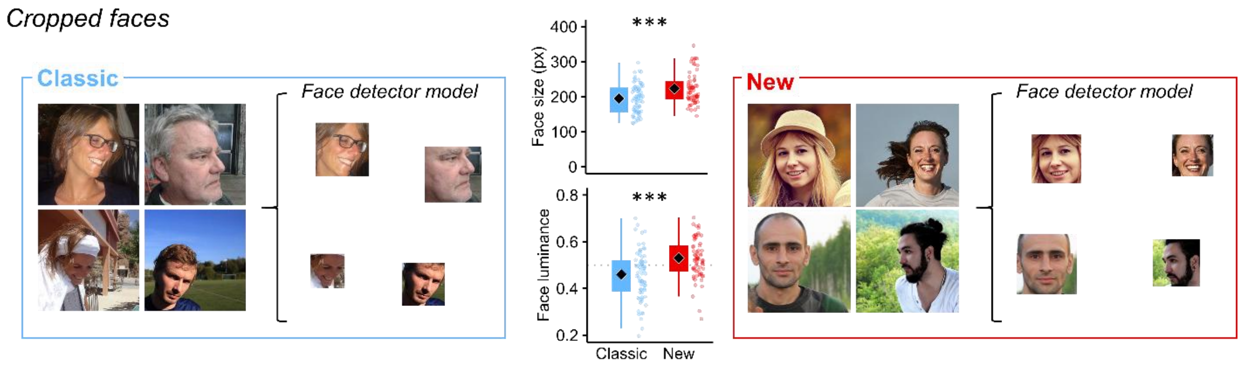

Figure S3. Size in pixel and luminance of the faces cropped from the background for both the Classic and the New sets. Black diamonds represent the mean. ***: $p_{FDR} < .001$.

Table S1. Mean, standard deviation and median values for the Face stimuli in full image and cropped around the face. All T-tests (t_{df}) were significant. Fisher tests (F) compare the variance across sets.

		Classic set	New set	Test	*p-value*
Full face images	***Luminance***				
	average	0.43	0.50	$\mathbf{t_{66} = 3.25}$	**.001**
	SD	0.12	0.13	$F = 1.17$	.26
	Median	0.43	0.52		
Face crop	***size (px)***				
	average	194.65	222.96	$\mathbf{t_{66} = 4.00}$	**.0002**
	SD	41.31	43.71	$F = 1.12$	.32
	median	194.5	212.5		
	Luminance				
	average	0.46	0.53	$\mathbf{t_{66} = 4.32}$	**< .0001**
	SD	0.11	0.08	$\mathbf{F = 1.60}$	**.028**
	median	0.46	0.53		

Supplementary method III – EEG acquisition

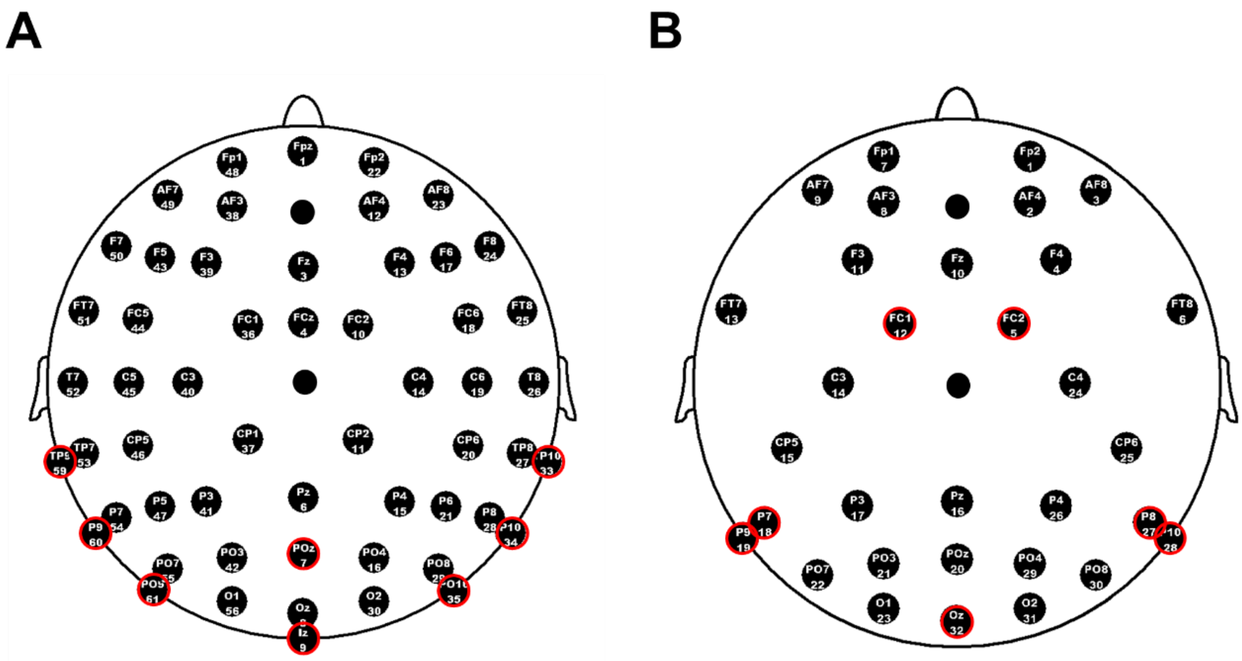

Figure S4. Electrode montage for the adult (A) and infant (B) EEG experiment (Experiment 1 and 2, respectively), with AFz as passive ground and Cz as reference. The electrodes marked in red are the ones excluded from the custom re-referencing because of their lack of antero-posterior counterpart.

Note on common vs custom average:

Considering our custom lay-out with a higher density of electrodes over posterior areas, a common average would artificially disequilibrate the strength of the signal towards the frontal areas by reducing the amplitude over the posterior electrodes. To circumvent this issue, a custom average was performed. It consisted in keeping in the average reference, only the electrodes which possess symmetrical counterparts across the midline. This custom average reference was then applied to all electrodes. This allows to adjust the signal more precisely to the actual strength of the neural activity engaged.

Supplementary results I – Experiment 1

Table S2. Results of the ANOVA on the amplitude of the general visual response from Experiment 1, in N = 19 adult participants. Significant effects are reported in red. SS: sum of square, df: degree of freedom, MS: mean square.

	SS	df	MS	*F*	*p*	η_p^2
Scrambling	0.95	1	0.95	1.51	0.24	0.08
Error	11.31	18	0.63			
Set	0.20	1	0.20	7.44	0.01	0.29
Error	0.49	18	0.03			
Scrambling × Set	0.14	1	0.14	1.18	0.29	0.06
Error	2.15	18	0.12			

Table S3. Results of the ANOVA on the amplitude of the face-selective response from Experiment 1, in N = 19 adult participants. Significant effects are reported in red. Mauchly's test estimated no violation of sphericity (unreported). SS: sum of square, df: degree of freedom, MS: mean square.

	SS	df	MS	*F*	*p*	η_p^2
Scrambling	182.25	1	182.25	108.19	.00	.86
Error	30.32	18	1.68			
Set	2.38	1	2.38	12.65	.00	.41
Error	3.39	18	0.19			
ROI	4.64	2	2.32	5.59	.01	.24
Error	14.94	36	0.42			
Scrambling × Set	2.16	1	2.16	14.83	.00	.45
Error	2.63	18	0.15			
Scrambling × ROI	5.32	2	2.66	8.38	.00	.32
Error	11.43	36	0.32			
Set × ROI	0.32	2	0.16	2.90	.07	.14
Error	1.96	36	0.05			
Scrambling × Set × ROI	0.38	2	0.19	3.31	.05	.16
Error	2.08	36	0.06			

Supplementary results II – Supporting evidence for potential overlap between 6th harmonic of 1 Hz and 1st harmonic of the 6-Hz response.

While our design does not allow us to disentangle the contribution of the 1-Hz response to the 6 Hz due to overlapping recording sites, several elements in our data suggest that the *Set* effect observed in the 6-Hz response (summed across 8 harmonics, **Table S2**) might result from the overlap between 6th harmonic of 1 Hz and 1st harmonic of the 6-Hz response.

First, inspection of the average data supports that the *Set* effect measured in the ANOVA was driven by the difference in the Intact condition, despite no interaction revealed by the ANOVA. Indeed, the advantage for the New Set reached marginal significance for Intact Classic vs. New (2.93 ± 0.38 < 3.12 ± 0.36 µV, t_{18} = 2.06, p = .05) but was clearly absent in the Scrambled condition (p = .84). Second, analyses on the general response separating values for each harmonic were performed. The analysis highlighted that the response in New set Intact was significantly higher than in all 3 other conditions (Intact Classic, Scrambled Classic and Scrambled New) for the 1st harmonic at 6 Hz only (Paired t-tests: t_{18}s > 2.45, p_{FDR} ≤ .05, **Figure S5**). All other comparisons (i.e., up to 48 Hz) were non-significant (ps > .11), except for Intact New (0.03 ± 0.006 µV) > Scrambled Classic (0.01 ± 0.004 µV) at the 6th harmonic (i.e., 36 Hz) which, however, did not survive the FDR correction (t_{18} = 2.59, p_{FDR} = .06). However, this effect appears negligible to our question as the 6th harmonic represents less than 1% of the summed response (i.e., between 0.38% and 0.85% across the 4 conditions). Finally, performing the ANOVA by summing only the values from the 2nd to the 8th harmonic (i.e., skipping the 1st harmonic) did not reveal an effect of *Set* (see **Table S4** below). Bayesian ANOVA provided moderate evidence against a main effect of *Set* (BF_{10} = 0.25, error = 0.99%) and strong evidence against a *Scrambling × Set* interaction (BF_{10} = 0.07, error = 4.4%), suggesting that summed responses across the harmonics 2–8 were similar across conditions and sets. Altogether these supporting evidence suggest that this effect was likely driven by the response at exactly 6 Hz, contaminated by the increase from the 1-Hz's 6th harmonic.

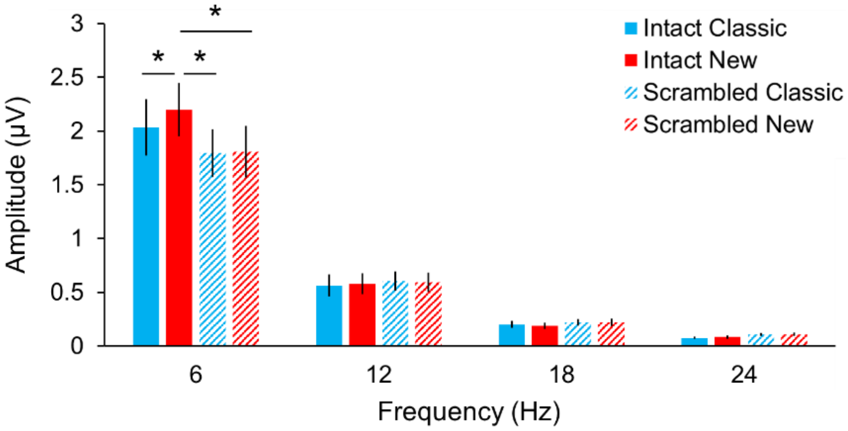

Figure S5. Investigating the differences of conditions on each harmonic of the general visual response. Amplitude (µV) for the 1st to 4th harmonic of the general visual response in all four conditions, over mO. *: p < .05 (FDR corrected), all other comparisons were non-significant. Error bars represent SEM.

Table S4. Results of the ANOVA on the amplitude of the general visual response in N = 19 adult participants, computed on the sum from 2nd to 8th (i.e., not including the 1st which overlaps with the 1-Hz response). No significant effects were found. SS: sum of square, df: degree of freedom, MS: mean square.

	SS	df	MS	*F*	*p*	η_p^2
Scrambling	0.16	1	0.16	1.61	.22	.082
Error	1.82	18	0.10			
Set	0.01	1	0.01	0.16	.70	.009
Error	0.55	18	0.03			
Scrambling × Set	0.00	1	0.00	0.09	.78	.005
Error	0.40	18	0.02			

Supplementary results III – Experiment 2

Table S5. Results of the ANOVA on the amplitude of the face-selective response from Experiment 2 in N = 46 infant participants. Significant effects are reported in red. Greenhouse-Geisser epsilon (ε) and corresponding adjusted p-values are reported when sphericity was violated (as estimated by Mauchly's test, unreported). SS: sum of square, df: degree of freedom, MS: mean square, GG: Greenhouse-Geisser, Adj: adjusted.

	SS	df	MS	*F*	*p*	η_p^2	GG ε	GG Adj. *p*
Age	1.16	1	1.16	0.31	.58	.01		
Error	164.91	44	3.75					
Set	15.88	1	15.88	6.97	.01	.14		
Set × Age	10.64	1	10.64	4.67	.04	.10		
Error	100.23	44	2.28					
ROI	2.43	2	1.21	0.87	.42	.02		
ROI × Age	3.82	2	1.91	1.37	.26	.03		
Error	122.34	88	1.39					
Set × ROI	2.36	2	1.18	0.80	.45	.02	.80	.43
Set × ROI × Age	0.85	2	0.43	0.29	.75	.01		
Error	129.41	88	1.47					

Table S6. Results of the ANOVA on the amplitude of the general visual response from Experiment 2 in N = 46 infant participants. Significant effects are reported in red. SS: sum of square, df: degree of freedom, MS: mean square.

	SS	df	MS	*F*	*p*	η_p^2
Age	31.39	1	31.39	4.55	.04	.09
Error	303.58	44	6.90			
Set	0.16	1	0.16	0.28	.60	.01
Set × Age	0.98	1	0.98	1.71	.20	.04
Error	25.26	44	0.57			

Table S7. Group-level *Z*-scores on the first four harmonics of the face-selective response in Experiment 2 (N = 46 infants) separately and summed. We display the 17 posterior electrodes ("post-elec"), the ROIs and the average over the scalp (N = 32 channels, "avg-elec"). Significance is highlighted for $p < .05$ (one-tailed).

	Classic set					***New set***				
	**1 Hz**	**2 Hz**	**3 Hz**	**4 Hz**	**Σ**	**1 Hz**	**2 Hz**	**3 Hz**	**4 Hz**	**Σ**
**CP5**	-0.59	-1.70	0.70	-0.65	-1.03	-0.81	**2.16***	-0.20	0.11	0.27
**CP6**	-0.47	-2.86	-1.61	0.20	-2.10	0.37	1.59	**2.73***	0.06	1.35
**P9**	-0.19	0.42	**1.99***	0.25	0.55	1.47	-0.18	-0.48	0.11	0.97
**P7**	**2.45***	-0.34	0.69	1.16	**2.73***	**3.42***	**2.38***	-0.07	1.71	**3.73***
**P3**	-0.99	0.81	0.60	0.55	-0.39	0.30	**2.70***	-1.12	**4.88***	**1.77***
**Pz**	0.22	0.72	0.83	0.52	0.74	1.37	-2.63	**1.77***	-0.16	0.83
**P4**	0.04	-1.43	0.13	0.63	-0.55	0.79	0.10	-0.74	0.55	0.49
**P8**	0.85	1.23	-0.95	0.54	1.00	**1.71***	**2.83***	-1.02	-0.42	**1.67***
**P10**	0.66	0.04	**1.72***	-0.14	0.92	**2.50***	1.29	**3.23***	1.19	**4.88***
**PO7**	1.08	1.12	1.26	**2.76***	**1.65***	**2.69***	**2.03***	1.41	**2.09***	**3.37***
**PO3**	-1.36	0.12	1.57	**2.57***	-0.40	-0.93	1.70	1.83	**2.00***	0.51
**POz**	-0.01	-1.28	0.09	-1.32	-0.74	0.71	-0.24	-0.27	0.81	0.41
**PO4**	-0.54	**2.00***	0.11	-0.76	0.19	-1.37	1.42	-1.34	-0.77	-0.90
**PO8**	-0.15	1.19	0.44	-3.59	-0.24	**2.41***	**2.02***	-0.08	**3.08***	**3.16***
**O1**	0.32	-1.09	-1.47	**3.09***	-0.22	**3.07***	-0.86	0.31	**2.45***	**2.90***
**Oz**	-0.48	0.89	0.99	1.56	0.46	**2.08***	0.26	0.91	**4.64***	**2.98***
**O2**	0.99	0.01	1.31	0.06	1.43	**2.41***	1.47	-0.64	**5.23***	**2.84***
N	1 (6%)	1 (6%)	2 (12%)	3 (18%)	2 (12%)	8 (47%)	7 (41%)	4 (24%)	8 (47%)	9 (53%)
lOT	0.78	0.26	**2.30***	1.26	1.20	**2.69***	**3.03***	-0.05	**3.46***	**3.61***
mO	-0.16	0.27	0.91	1.52	0.44	**2.06***	0.52	0.98	**4.15***	**3.08***
rOT	0.32	-0.70	-0.06	-0.97	0.32	**2.95***	**3.33***	1.32	1.20	**3.62***
post-elec	0.32	-0.08	1.33	1.10	0.61	**3.42***	**3.41***	1.04	**4.11***	**5.33***
avg-elec	1.10	-0.43	**1.76***	1.09	1.11	**2.80***	**3.96***	1.00	**3.57***	**5.10***

Table S8. Z-scores according to younger vs older infants (following the median split) and *Set*. We display the 17 posterior electrodes ("post-elec"), the ROIs and the average over the scalp (N = 32 channels, "avg-elec"). Significance is highlighted for $p < .05$ (one-tailed).

	Younger half		***Older half***	
	Classic	***New***	***Classic***	***New***
CP5	-1.20	0.74	-0.41	-0.51
CP6	-1.03	**1.96***	-1.82	0.48
P9	-0.42	0.22	1.56	1.53
P7	0.59	**2.25***	**3.72***	**2.45***
P3	-1.28	**1.65***	1.02	1.10
Pz	0.53	1.24	0.58	0.12
P4	-0.04	0.15	-1.16	0.74
P8	-0.63	1.04	**2.35***	1.21
P10	-0.74	**2.65***	**2.86***	**3.72***
PO7	0.72	**3.08***	**1.81***	**2.75***
PO3	-0.85	**1.71***	0.51	-0.62
POz	-0.51	0.16	-0.17	0.40
PO4	-0.33	0.12	0.96	-1.16
PO8	-0.77	1.27	0.35	**3.00***
O1	0.28	**3.53***	-0.75	**1.71***
Oz	0.56	**3.35***	0.25	1.01
O2	0.59	**2.05***	1.48	**2.08***
N	0 (0%)	9 (53%)	4 (24%)	6 (35%)
lOT	-0.46	**3.11***	**3.27***	**2.86***
mO	-0.05	**2.97***	0.82	1.40
rOT	-1.09	**2.26***	**1.75***	**3.07***
post-elec	-0.64	**4.54***	**2.20***	**3.94***
avg-elec	-0.24	**3.90***	**2.57***	**3.17***

Supplementary results IV - Inter-set correlations

To assess if the responses from both sets were related, we calculated the inter-set correlations for both responses in both adult and infant experiments.

In adults (Experiment 1), the inter-set correlation for the face-selective response was high (r = .86; **Figure S6, Table S9**), indicating that participants who responded e.g., strongly to one set tended to respond strongly to the other. This inter-set correlation did not differ significantly from the split-half correlations within each set (Classic: r = .76, Z = 0.83, p = .41; New: r = .93, Z = 0.99, p = .32), suggesting that the inter-set relationship approaches the reliability ceiling imposed by measurement noise. Moreover, as mentioned in the main Results section, the split-half correlations for Classic and New were themselves not significantly different (Z = 1.82, p = .06), confirming comparable internal consistency across sets. Notably, the ANOVA revealed a large Set effect (see main results of Experiment 1), indicating that the mean response differed substantially between Classic and New sets. These results highlight that, despite the robust mean difference, the high inter-set correlation demonstrates strong participant-level consistency: individuals' responses remained largely consistent across sets, with the set differences reflecting systematic, group-level effects.

In infants (Experiment 2), by contrast, inter-set correlations were high for the general visual response (r = .86) but not significant for the face-selective response (r = .20, p = .18; **Figure S6**, **Table S9**). This pattern is consistent with the ANOVA results showing stimulus-dependent selectivity in infants. The absence of correlation in infants, although potentially due to an only weak response in the Classic set, reinforces the need for tailored stimuli to accurately assess early face-selective neural responses and demonstrates that using the New set reveals latent face-selectivity that the Classic set fails to capture.

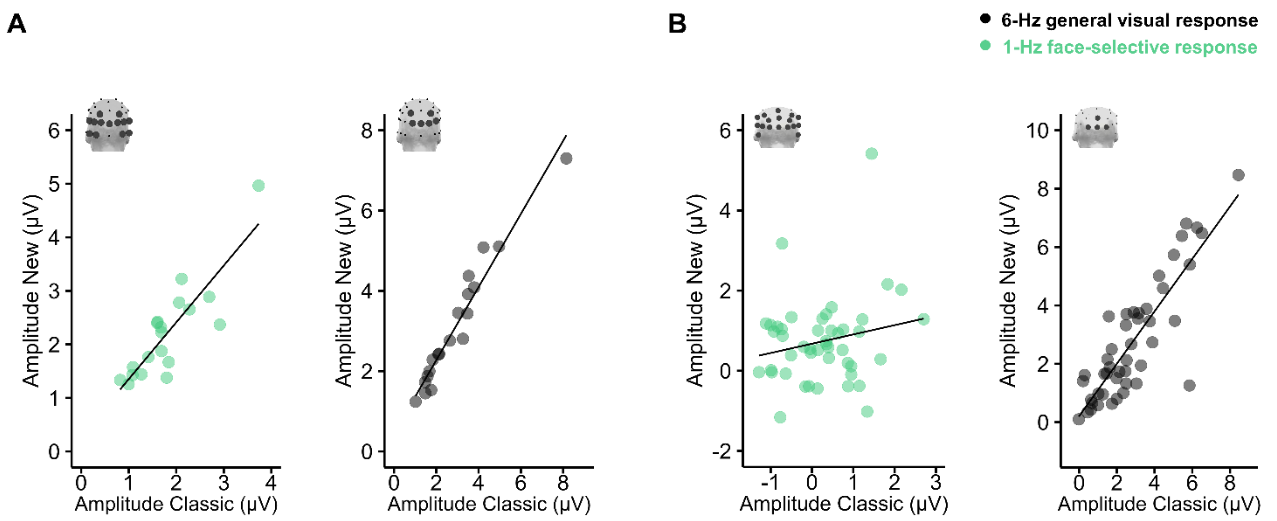

Figure S6. Inter-set correlation for Experiment 1 in adults (**A**) and Experiment 2 in infants (**B**) for the face-selective (green) and general visual responses (black). The amplitude obtained in response to the Classic set is plotted as a function of the amplitude in response to the New set over the average of the ROIs, as illustrated from the electrode maps above each graph.

Table S9. Pearson correlation coefficients (R) and significance testing for the inter-set correlations presented in Figure S6. Significance is highlighted in bold for *p* < .05 (one-tailed).

	Experiment 1 (N = 19 adults)		***Experiment 2 (N = 46 infants)***	
	Face response	General response	Face response	General response
R	**.86**	**.97**	.20	**.86**
t	**6.90**	**16.88**	1.36	**10.97**
p	**.00**	**.00**	.18	**.00**
